# Body Weight Changes and Exploratory Metabolic Outcomes During Semaglutide and Tirzepatide Treatment: A Real-World Longitudinal Cohort Study from Türkiye

**DOI:** 10.3390/jcm15207876

**Published:** 2026-10-13

**Authors:** Esra Çokiçli, Murat Kayır, Alp Russel Abbasoğlu, Hanise Ozkan Sonay, İrem Kıraç Utku

**Affiliations:** 1 Department of Internal Medicine, Istanbul Medipol University, 34810 Istanbul, Türkiye; kayirmurat@gmail.com (M.K.); alp.abbasoglu@std.medipol.edu.tr (A.R.A.); 2 Department of Internal Medicine, Kanuni Sultan Suleyman Training and Research Hospital, 34303 Istanbul, Türkiye; haniseozkan@hotmail.com; 3 Department of Internal Medicine, Division of Geriatrics, Tekirdag Dr. Ismail Fehmi Cumalioglu City Hospital, 59030 Tekirdag, Türkiye; driremkirac@gmail.com

**Keywords:** obesity, body weight, weight loss, glucagon-like peptide-1 receptor agonists, semaglutide, tirzepatide, retrospective studies, longitudinal studies

## Abstract

**Background/Objectives:** Comparative real-world evidence on early body-weight changes during semaglutide and tirzepatide treatment remains limited, particularly in populations with unequal and irregularly timed follow-up. To compare longitudinal percentage changes in body weight between adults initiating semaglutide and those initiating tirzepatide and to describe changes in anthropometric measures, weight-loss responses, and exploratory metabolic outcomes. **Methods:** This retrospective, single-center, real-world longitudinal cohort study included adults aged ≥18 years initiating semaglutide or tirzepatide for weight management, with a body mass index (BMI) ≥30 kg/m^2^ or ≥27 kg/m^2^ with a weight-related comorbidity. Patients with type 2 diabetes and concomitant obesity were eligible. Participants were naïve to incretin-based therapy; pregnancy, previous bariatric surgery, and active malignancy were exclusion criteria. Anthropometric and laboratory data were extracted from clinical records. Percentage body-weight change over days 1–230 was analyzed using a linear mixed-effects model incorporating exact follow-up time and adjusting for age, sex, baseline BMI, and diabetes status. Scheduled-visit and sensitivity analyses were also performed. **Results:** The cohort included 184 patients: 94 (51.1%) receiving semaglutide and 90 (48.9%) receiving tirzepatide. Overall, mean age was 41.7 ± 12.0 years, baseline weight was 97.0 ± 17.9 kg, and 139 patients (75.5%) were female. The longitudinal analysis included 345 measurements from 148 patients (80.4%). The treatment-by-time interaction was significant (*p* = 0.002). Adjusted weight changes with semaglutide versus tirzepatide were −3.39% versus −4.44% at month 1 (*p* = 0.116), −8.38% versus −11.52% at month 3 (*p* < 0.001), and −14.40% versus −15.06% at month 6 (*p* = 0.699). Sensitivity analyses yielded consistent findings. Only four tirzepatide-treated patients had measurements within the month 6 visit window; therefore, the month 6 estimate for this group relied primarily on the model and requires cautious interpretation. **Conclusions:** Both semaglutide and tirzepatide were associated with early reductions in body weight. Tirzepatide was associated with larger model-adjusted weight loss at month 3 (between-group difference, −3.14 percentage points; 95% CI, −4.55 to −1.74; *p* < 0.001), whereas no statistically significant between-group difference was observed at month 1 (*p* = 0.116). Only four tirzepatide-treated patients had measurements within the month 6 visit window, limiting the reliability of this comparison. Longer-term comparisons require cohorts with more balanced follow-up and more observations at later time points.

## 1. Introduction

Obesity is a chronic, progressive, and relapsing disease that has become a major global health challenge [1]. Between 1990 and 2022, the age-standardized prevalence of obesity more than doubled among women and nearly tripled among men worldwide, with more than one billion people estimated to be living with obesity in 2022 [2]. In Türkiye, the Turkish Statistical Institute’s 2025 Türkiye Health Survey reported an obesity prevalence of 21.8% among individuals aged 15 years and older [3]. Looking ahead, the World Obesity Atlas 2025 projects that approximately 1.13 billion adults aged ≥20 years worldwide will be living with obesity by 2030. For Türkiye, the projected numbers across BMI categories was ≥30 kg/m^2^ among adults aged ≥20 years sum to approximately 24.8 million by 2030. These model-based projections are distinct from the national survey prevalence reported above and indicate a substantial future burden of obesity [4]. Beyond excess body weight, obesity is associated with type 2 diabetes mellitus, cardiovascular disease, metabolic dysfunction-associated steatotic liver disease, and increased mortality [1]. Accordingly, effective and sustainable weight-management strategies are needed to reduce both the clinical and public-health burden of obesity.

Incretin-based therapies have substantially changed the pharmacological management of obesity. Semaglutide is a long-acting glucagon-like peptide-1 receptor agonist, whereas tirzepatide is a dual glucose-dependent insulinotropic polypeptide and glucagon-like peptide-1 receptor agonist. In the STEP 1 trial, once-weekly semaglutide 2.4 mg produced a mean body-weight reduction of 14.9% at 68 weeks among adults with overweight or obesity without diabetes [5]. In the SURMOUNT-1 trial, tirzepatide produced dose-dependent mean weight reductions of 15.0–20.9% at 72 weeks [6]. More recently, the head-to-head SURMOUNT-5 trial demonstrated larger reductions in body weight and waist circumference with tirzepatide than with semaglutide among adults with obesity without diabetes [7]. In addition to weight reduction, semaglutide has been shown to reduce the risk of major adverse cardiovascular events in adults with overweight or obesity and established cardiovascular disease but without diabetes, further supporting the clinical relevance of incretin-based obesity treatment [8].

Although randomized trials provide strong evidence of efficacy under controlled conditions, their findings may not fully reflect routine clinical practice. Real-world treatment is influenced by variation in dose escalation, medication availability, treatment persistence, switching, comorbidities, and the timing and frequency of follow-up assessments. A large US electronic health record study found that tirzepatide was associated with larger weight loss than semaglutide at 3, 6, and 12 months, although both treatments produced clinically meaningful reductions in body weight [9]. Similarly, the SHAPE study showed substantial 1-year weight loss among persistent users of semaglutide 2.4 mg and tirzepatide without type 2 diabetes [10]. However, real-world studies remain vulnerable to confounding by treatment selection, calendar time, treatment persistence, and unequal follow-up opportunity. Comparative real-world studies have primarily focused on body weight, while inflammatory markers and derived visceral adiposity indices have received less attention [9,10,11].

Real-world comparative evidence from Türkiye remains limited. A recent single-center study of 169 adults with overweight or obesity without diabetes compared liraglutide, semaglutide, and tirzepatide over 36 weeks and reported significant reductions in body weight and waist circumference with all three treatments, with larger weight loss in the tirzepatide group [11]. Nevertheless, further evidence is needed in broader routine-care populations, including patients with and without type 2 diabetes, particularly from studies using longitudinal methods that account for irregularly timed measurements and unequal follow-up opportunity. This is especially relevant during the early treatment period, when differences in calendar-time initiation patterns may substantially affect the availability of later follow-up observations.

The primary aim of this study was to compare longitudinal percentage changes in body weight between patients initiating semaglutide and those initiating tirzepatide under real-world clinical conditions. Secondary aims were to describe changes in anthropometric measures and clinically relevant weight-loss responses at scheduled follow-up visits. Changes in metabolic, hepatic, inflammatory, and derived visceral adiposity markers were evaluated as exploratory outcomes.

## 2. Materials and Methods

### 2.1. Study Design and Setting

This retrospective, single-center, longitudinal observational study was conducted at the Internal Medicine Department of Istanbul Medipol University, Istanbul, Türkiye. The medical records of consecutive adults who initiated semaglutide or tirzepatide for weight management between 24 October 2024 and 7 March 2026 were reviewed. Clinical and laboratory data generated during routine care were retrospectively collected. The data cut-off date was 10 March 2026.

The study was conducted in accordance with the principles of the Declaration of Helsinki and was approved by the Non-Invasive Clinical Research Ethics Committee of Istanbul Medipol University (approval date: 17 March 2026; decision number: E-10840098-50.04-2068). Because of the retrospective design and use of routinely collected clinical data, the requirement for written informed consent was waived by the ethics committee.

### 2.2. Study Population and Treatment-Group Definition

Patients were eligible if they were aged ≥18 years, had a BMI ≥30 kg/m^2^ or a BMI ≥27 kg/m^2^ with at least one weight-related comorbidity, and initiated semaglutide or tirzepatide specifically for weight management during the study period. Patients with type 2 diabetes mellitus were also eligible, provided that they had concomitant obesity and treatment was initiated for weight management. All participants were naïve to glucagon-like peptide-1 receptor agonist or dual glucose-dependent insulinotropic polypeptide/glucagon-like peptide-1 receptor agonist therapy at baseline. Patients who were pregnant, had a history of bariatric surgery, or had active malignancy were excluded. An available baseline body-weight measurement was required.

The index date was defined as the documented date of semaglutide or tirzepatide initiation. Treatment groups were defined according to the medication initiated on the index date.

The primary longitudinal analytic cohort comprised patients with at least one valid follow-up body-weight measurement obtained between days 1 and 230 after treatment initiation. Patients without a valid follow-up body-weight measurement during this period contributed to the description of the overall baseline cohort but were not included in the primary mixed-effects analysis.

All treatment decisions and follow-up visits were managed by the same physician in the Internal Medicine Department. There was no standardized local treatment protocol. Treatment selection and dosing were individualized according to clinical assessment, treatment response, and tolerability, consistent with the principles of individualized obesity management described in contemporary guidance [12,13]. Dose escalation was gradual and performed exclusively under physician supervision; patients did not independently increase their doses. Doses were maintained when appetite was adequately controlled and weight loss continued. Adverse effects were monitored during follow-up.

### 2.3. Data Collection and Clinical Definitions

Demographic, anthropometric, clinical, and laboratory data were extracted retrospectively from electronic medical records. Baseline variables included age, sex, height, body weight, BMI, waist circumference, hip circumference, waist-to-hip ratio, diabetes mellitus, hypertension, hyperlipidemia, and atherosclerotic cardiovascular disease. Baseline laboratory variables included glycated hemoglobin (HbA1c), fasting glucose, fasting insulin, creatinine, alanine aminotransferase (ALT), aspartate aminotransferase (AST), gamma-glutamyl transferase (GGT), C-reactive protein (CRP), erythrocyte sedimentation rate (ESR), total cholesterol, low-density lipoprotein (LDL) cholesterol, high-density lipoprotein (HDL) cholesterol, and triglycerides, when available.

Body mass index (BMI) was calculated using the equation:

BMI (kg/m^2^) = body weight (kg)/[height (m)]^2^.

Waist-to-hip ratio (WHR) was calculated using the equation:

WHR = waist circumference (cm)/hip circumference (cm).

Both circumferences were expressed in centimeters; therefore, WHR is a dimensionless ratio.

Diabetes mellitus, hypertension, hyperlipidemia, and atherosclerotic cardiovascular disease were identified from the diagnoses documented in the medical records at baseline. Diabetes status was assessed using documented diagnoses and available baseline fasting glucose and HbA1c results. Oral glucose tolerance testing was not routinely performed in patients without a previous diabetes diagnosis.

All anthropometric and laboratory measurements were obtained as part of routine clinical care. Consequently, the timing and availability of follow-up measurements were not standardized and varied among participants. No additional measurements were performed specifically for this study.

### 2.4. Follow-Up Measurements and Scheduled-Visit Windows

In addition to the primary longitudinal analysis using the exact timing of all available follow-up measurements, a separate planned-visit analysis was performed to provide clinically interpretable summaries at nominal follow-up time points. Because follow-up visits in routine clinical practice did not occur at fixed intervals, predefined visit windows were used to accommodate variability in visit timing while preserving clinically meaningful follow-up periods. A narrower window was used for early follow-up, whereas symmetric ±30-day windows were used around the nominal month 3 and month 6 time points to accommodate higher variability in routine-care scheduling. Accordingly, month 1, month 3, and month 6 visits were defined as days 15–45, 60–120, and 150–210 after treatment initiation, respectively. These nonoverlapping windows prevented a measurement from contributing to more than one planned visit. When more than one measurement was available within a given window, the measurement closest to the nominal target day—day 30, day 90, or day 180—was selected to minimize variation in elapsed treatment time. In the event of equal proximity, the later measurement was used.

The scheduled-visit windows were used only for supportive analyses of anthropometric changes, weight-loss thresholds, and month 3 laboratory outcomes. In contrast, the primary longitudinal mixed-effects analysis included all valid follow-up body-weight measurements obtained between days 1 and 230, including measurements that did not fall within a scheduled-visit window.

Potential follow-up duration was calculated from the documented treatment initiation date to the data cut-off date of 10 March 2026. Patients with at least 150 days between treatment initiation and the data cut-off date were considered to have had the opportunity to enter the month 6 follow-up window. This definition represented a follow-up opportunity and did not require the presence of an actual month 6 measurement.

### 2.5. Study Outcomes

The primary outcome was percentage change in body weight from baseline during the first 230 days after treatment initiation. Percentage body-weight change was calculated as

[(follow-up body weight − baseline body weight)/baseline body weight] × 100.

The primary analysis compared the longitudinal trajectories of percentage body-weight change between the semaglutide and tirzepatide groups using all valid repeated measurements obtained during this period.

Secondary outcomes included within-participant changes in body weight, BMI, waist circumference, hip circumference, and waist-to-hip ratio at the scheduled month 1, month 3, and month 6 visits. Clinically relevant weight-loss responses were defined as reductions of at least 5%, 10%, 15%, and 20% from baseline and were evaluated separately at each scheduled visit.

Exploratory outcomes included within-participant changes from baseline to month 3 in glycemic variables, lipid parameters, hepatic enzymes, inflammatory markers, and derived metabolic and visceral adiposity indices. These outcomes were evaluated using available paired measurements and were not intended to estimate comparative treatment effects between semaglutide and tirzepatide.

### 2.6. Derived Metabolic and Visceral Adiposity Indices

The following indices were selected as complementary, routinely calculable surrogate measures of insulin resistance, plasma atherogenicity, and adiposity-related metabolic dysfunction. All were evaluated as exploratory outcomes.

HOMA-IR was calculated as a fasting surrogate of insulin resistance using the original homeostasis model assessment equation [14]:

HOMA-IR = [FI × FG]/405,

where FI is fasting insulin in µIU/mL and FG is fasting glucose in mg/dL. The denominator 405 corresponds to the original normalization constant of 22.5 when glucose is expressed in mmol/L, multiplied by the glucose conversion factor of 18. HOMA-IR assumes a basal fasting steady state between glucose and insulin and is reported as a unitless index.

The triglyceride-glucose (TyG) index was selected as a non-insulin-based surrogate of insulin resistance and calculated as follows [15].

TyG = ln[(TG × FG)/2],

where TG and FG are fasting triglyceride and glucose concentrations, respectively, both expressed in mg/dL. Here, ln denotes the natural logarithm, and 2 is the fixed scaling constant in the published equation. The calculation uses the numerical values in these specified units.

The atherogenic index of plasma (AIP) was selected to describe the triglyceride–HDL cholesterol balance associated with plasma atherogenicity [16]:

AIP = log_10_(TG/HDL-C),

where TG and HDL-C are triglyceride and high-density lipoprotein cholesterol concentrations, respectively, both expressed in mmol/L. The base-10 logarithm is applied to their molar concentration ratio; AIP is dimensionless.

Lipid accumulation product (LAP) was selected as a surrogate of lipid overaccumulation, combining abdominal size and triglyceride concentration [17].

Men: LAP = (WC − 65) × TG.

Women: LAP = (WC − 58) × TG.

Here, WC is waist circumference in cm, and TG is fasting triglyceride concentration in mmol/L. The constants 65 and 58 cm represent the sex-specific estimated minimum waist circumferences used in the original derivation. LAP is expressed in cm·mmol/L.

The visceral adiposity index (VAI) was selected as an indirect indicator of visceral adipose dysfunction incorporating anthropometric and lipid measurements [18]:

Men: VAI = [WC/(39.68 + 1.88 × BMI)] × (TG/1.03) × (1.31/HDL-C).

Women: VAI = [WC/(36.58 + 1.89 × BMI)] × (TG/0.81) × (1.52/HDL-C).

WC is expressed in cm, BMI in kg/m^2^, and TG and HDL-C in mmol/L. The constants 39.68 and 36.58 and coefficients 1.88 and 1.89 define the sex-specific BMI-based reference waist circumference in cm. The lipid constants 1.03, 0.81, 1.31, and 1.52 are expressed in mmol/L. These published coefficients were retained without re-estimation; VAI is dimensionless.

TyG-BMI and TyG–waist circumference (TyG-WC) were selected to combine the TyG index with overall and abdominal adiposity, respectively [15].

TyG-BMI = TyG × BMI.

TyG-WC = TyG × WC.

BMI was expressed in kg/m^2^ and WC in cm. The resulting composite scores retain the corresponding anthropometric unit conventions.

These calculations require fasting biochemical measurements and the specified input units; logarithmic calculations additionally require positive input values. The indices were interpreted as calculated surrogates rather than direct measurements of insulin sensitivity, visceral fat volume, or cardiovascular outcomes.

### 2.7. Statistical Analysis

Statistical analyses were performed using IBM SPSS Statistics for Windows, version 28.0 (IBM Corp., Armonk, NY, USA). Joint two-degree-of-freedom Wald chi-square tests for the overall treatment-by-time interaction were calculated from the fixed-effect estimates and their corresponding covariance matrix obtained from the mixed-effects models. The distributions of continuous variables were evaluated using histograms, Q–Q plots, and the Shapiro–Wilk test. Normally distributed continuous variables were summarized as mean ± standard deviation (SD), whereas non-normally distributed variables were presented as median and interquartile range (first to third quartiles, Q1–Q3). Categorical variables were summarized as numbers and percentages.

Baseline characteristics were compared between the semaglutide and tirzepatide groups. Normally distributed continuous variables were compared using Welch’s independent-samples *t*-test, whereas non-normally distributed continuous variables were compared using the Mann–Whitney U test. Categorical variables were compared using Pearson’s chi-square test or Fisher’s exact test, as appropriate. Missing observations were excluded on a variable-by-variable basis, and the number of available observations was reported for variables with incomplete data.

Supportive planned-visit analyses used the predefined month 1, month 3, and month 6 windows and measurement-selection rules described in Section 2.4. The primary mixed-effects model included all valid follow-up body-weight measurements obtained between days 1 and 230, including measurements outside these windows.

Changes in body weight, BMI, waist circumference, hip circumference, and waist-to-hip ratio at the scheduled month 1, month 3, and month 6 visits were evaluated among participants with both baseline and corresponding follow-up measurements. Within-participant changes were calculated as the follow-up value minus the baseline value. After evaluation of the distributions of the change scores, these changes were analyzed using paired-samples *t*-tests and reported as mean changes with 95% confidence intervals (CIs). Cohen’s d_z was calculated as the standardized effect size for paired measurements. Because the number and composition of participants differed across the scheduled visits, unadjusted changes at months 1, 3, and 6 were not directly compared with one another.

The proportions achieving the weight-loss thresholds defined in Section 2.5 were compared between treatment groups using Pearson’s chi-square test or Fisher’s exact test, as appropriate. No statistical comparison was performed when no participant in either treatment group achieved the specified threshold. These scheduled-visit comparisons were considered supportive and unadjusted analyses.

Changes in metabolic, hepatic, visceral adiposity, and inflammatory markers between baseline and month 3 were evaluated among participants with paired measurements. Because these variables and their within-participant changes were generally non-normally distributed, comparisons were performed using the Wilcoxon signed-rank test. Results were presented as medians (Q1–Q3), together with the Wilcoxon W statistic and nominal *p* value. Outcomes with very small numbers of paired observations were reported separately in the Supplementary Material and interpreted descriptively.

The primary longitudinal analysis evaluated percentage change in body weight using all valid follow-up measurements obtained between days 1 and 230 after treatment initiation. A linear mixed-effects model with a patient-specific random intercept was fitted to account for within-participant correlation, irregularly timed observations, and unequal numbers of repeated measurements.

Exact time elapsed since treatment initiation was expressed in months and modeled using linear and quadratic terms to accommodate a nonlinear weight-loss trajectory. Fixed effects included linear and quadratic terms for time, treatment group defined according to the medication initiated at baseline, treatment-by-time and treatment-by-time-squared interactions, age, sex, baseline BMI, and diabetes status. Model parameters were estimated using restricted maximum likelihood. Model-adjusted mean percentage changes and their 95% CIs were estimated at months 1, 3, and 6. Between-group contrasts were calculated as tirzepatide minus semaglutide; therefore, negative values indicated a larger model-estimated weight reduction with tirzepatide. The joint contribution of the treatment-by-time and treatment-by-time-squared interaction terms was evaluated using a two-degree-of-freedom Wald chi-square test. Multicollinearity among the prespecified baseline covariates was assessed using variance inflation factors (VIFs), with values below 5 considered to indicate the absence of substantial multicollinearity.

Linear and quadratic time terms were selected to allow the rate of weight loss to change during follow-up while maintaining a parsimonious model appropriate for the available repeated measurements. A patient-specific random intercept was used because the number and timing of measurements varied substantially among participants and the sparse longer-term data did not support reliable estimation of a more complex random-effects structure.

As a sensitivity analysis, follow-up time was modeled categorically using measurements selected within the scheduled month 1, month 3, and month 6 visit windows. The model included treatment group, categorical time, treatment-by-time interactions, age, sex, baseline BMI, and diabetes status, together with a patient-specific random intercept. This analysis was performed to determine whether the principal findings were dependent on modeling time using linear and quadratic terms.

As an additional sensitivity analysis addressing the difference in calendar-time treatment initiation between the groups, treatment initiation date was added to the primary mixed-effects model as a continuous calendar-time covariate, expressed in months and centered at the mean treatment initiation date of the analytic cohort. All other fixed- and random-effect specifications were unchanged, and the same analytic dataset was used.

To evaluate differences in follow-up opportunity between the treatment groups, treatment initiation dates and potential follow-up durations, defined in Section 2.4, were summarized and compared.

No single imputation or last-observation-carried-forward procedure was used. Scheduled-visit analyses were conducted using available paired observations. The mixed-effects models incorporated all available valid repeated measurements and provided estimates under the assumption that missing observations were missing at random, conditional on the variables included in the models.

Because this was a retrospective real-world cohort study including all eligible patients treated during the predefined study period, no a priori sample-size calculation was performed. Post hoc power analysis was not conducted because it provides limited additional information once effect estimates and their confidence intervals are available. Statistical precision was therefore evaluated using 95% CIs.

All statistical tests were two-sided, and *p* < 0.05 was considered statistically significant. No formal adjustment for multiple comparisons was applied. Accordingly, secondary laboratory outcomes, derived metabolic indices, individual weight-loss thresholds, and inflammatory-marker analyses were considered exploratory and hypothesis-generating.

## 3. Results

### 3.1. Study Population and Analytic Cohort

**Figure 1:**
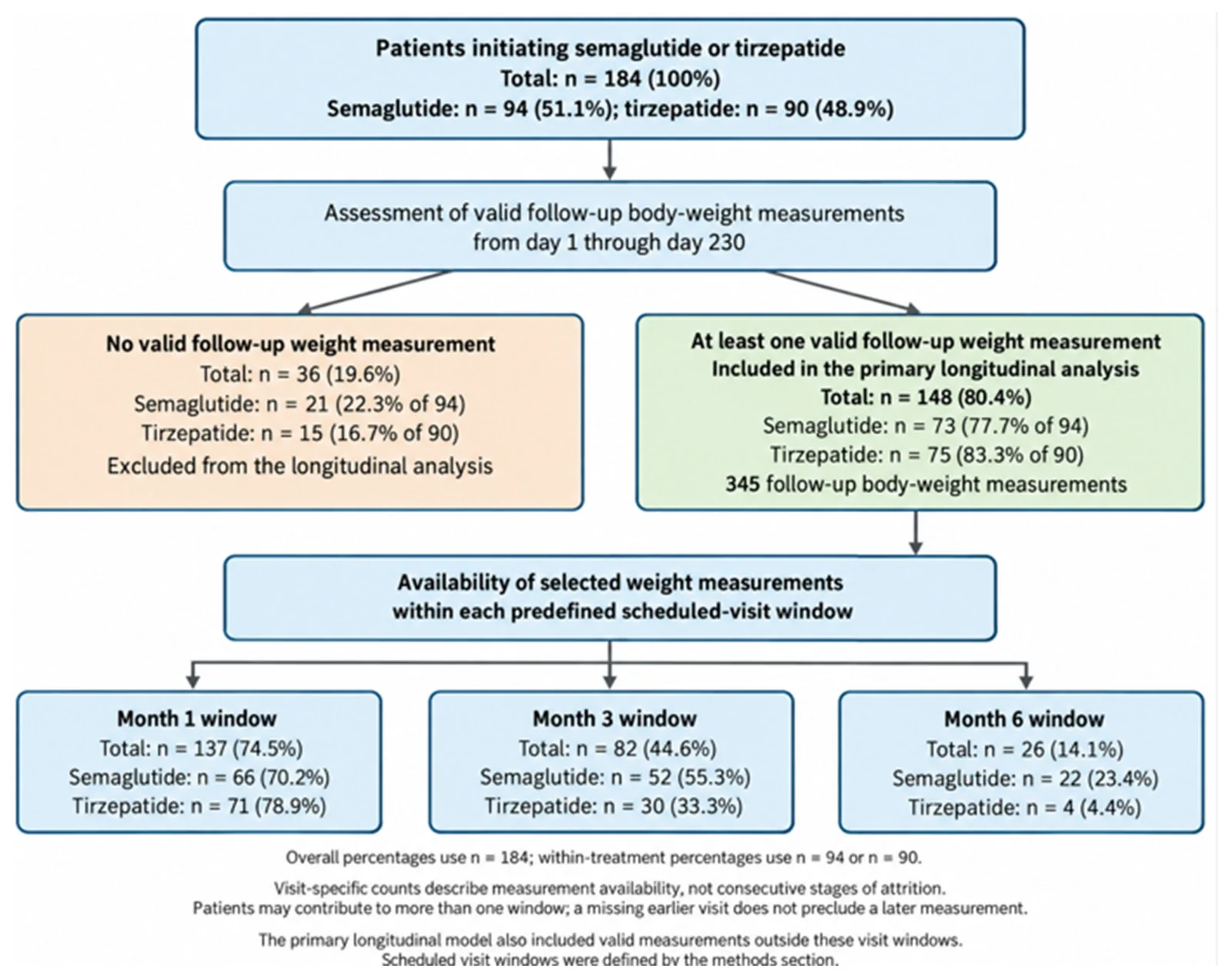
Study flow and primary analytic cohort. Percentages for treatment allocation and overall analytic inclusion are based on the total cohort (*n* = 184); percentages within each treatment group are based on the respective baseline group size. The analytic cohort contributed 345 follow-up body-weight measurements. Scheduled-visit counts indicate measurement availability within each predefined window rather than consecutive stages of attrition; individual patients could contribute measurements to more than one window.

A total of 184 patients were included, of whom 94 (51.1%) initiated semaglutide and 90 (48.9%) initiated tirzepatide. Overall, 23 patients (12.5%) had concomitant type 2 diabetes mellitus. The primary longitudinal analysis included 148 patients (80.4%) with at least one valid follow-up body-weight measurement obtained between days 1 and 230: 73 of 94 semaglutide-treated patients (77.7%) and 75 of 90 tirzepatide-treated patients (83.3%). The remaining 36 patients (19.6%), comprising 21 semaglutide-treated patients (22.3% of that group) and 15 tirzepatide-treated patients (16.7% of that group), had no valid follow-up measurement and were excluded from the longitudinal analysis. The included patients contributed 345 follow-up measurements (Figure 1).

Of the 184 patients who initiated semaglutide or tirzepatide, 148 had at least one valid follow-up body-weight measurement obtained between days 1 and 230 and were included in the primary longitudinal analysis. These patients contributed 345 follow-up measurements. Treatment groups were defined according to the medication initiated at baseline.

Treatment initiation occurred significantly later in the tirzepatide group. Among all 184 patients, the median treatment initiation date was 22 September 2025 (Q1–Q3, 27 July–3 December 2025) in the semaglutide group and 24 November 2025 (Q1–Q3, 19 October–30 December 2025) in the tirzepatide group (U = 2562.0, *p* < 0.001). Correspondingly, the median potential follow-up duration by the data cut-off date was 169 days (Q1–Q3, 98–225) for semaglutide and 108 days (Q1–Q3, 71–141) for tirzepatide (U = 2562.0, *p* < 0.001). At least 150 days of potential follow-up were available for 52 of 94 patients (55.3%) in the semaglutide group and 14 of 90 patients (15.6%) in the tirzepatide group (χ^2^ = 31.60, *p* < 0.001). Thus, the limited number of tirzepatide observations at month 6 should be interpreted in the context of the later calendar-time initiation of tirzepatide and the substantially shorter opportunity for longer-term follow-up (Table 1). The decrease in scheduled-visit counts should not be interpreted solely as loss to follow-up. Later treatment initiation limited the opportunity to reach the month 6 window before the data cut-off, particularly in the tirzepatide group, while irregular assessment timing also affected measurement availability within the predefined windows. Individual reasons for unavailable measurements were not systematically recorded.

### 3.2. Baseline Characteristics

The mean age of the study population was 41.7 ± 12.0 years, and 139 patients (75.5%) were female. Mean baseline body weight and BMI were 97.0 ± 17.9 kg and 35.00 ± 5.30 kg/m^2^, respectively. Mean waist circumference was 105.6 ± 13.9 cm, and mean waist-to-hip ratio was 0.871 ± 0.090. Type 2 diabetes mellitus was present in 23 patients (12.5%), hypertension in 17 (9.2%), hyperlipidemia in 8 (4.3%), and atherosclerotic cardiovascular disease in 3 (1.6%) (Table 1).

Baseline demographic, anthropometric, and clinical characteristics were broadly comparable between the treatment groups. Mean age was 40.4 ± 12.1 years in the semaglutide group and 43.1 ± 11.7 years in the tirzepatide group (*p* = 0.127). Corresponding baseline body weights were 96.3 ± 18.5 and 97.7 ± 17.3 kg (*p* = 0.611), and baseline BMI values were 34.63 ± 5.66 and 35.38 ± 4.90 kg/m^2^, respectively (*p* = 0.336). No statistically significant between-group differences were observed in sex distribution, waist or hip circumference, waist-to-hip ratio, or the prevalence of diabetes, hypertension, hyperlipidemia, or atherosclerotic cardiovascular disease (all *p* > 0.05) (Table 1).

Baseline glycemic, lipid, hepatic, inflammatory, and derived metabolic variables were also similar between the groups. Median HbA1c was 5.80% (Q1–Q3, 5.50–6.20) in the semaglutide group and 5.70% (5.40–6.07) in the tirzepatide group (*p* = 0.208). Median fasting glucose was 100.0 (93.8–113.0) versus 100.5 (94.0–105.8) mg/dL (*p* = 0.444), and median HOMA-IR was 3.88 (2.64–6.93) versus 3.98 (2.85–6.43), respectively (*p* = 0.953). No statistically significant baseline differences were identified in LDL cholesterol, HDL cholesterol, triglycerides, TyG index, AIP, liver enzymes, CRP, or erythrocyte sedimentation rate (Supplementary Table S1).

### 3.3. Primary Longitudinal Analysis of Body-Weight Change

**Figure 2:**
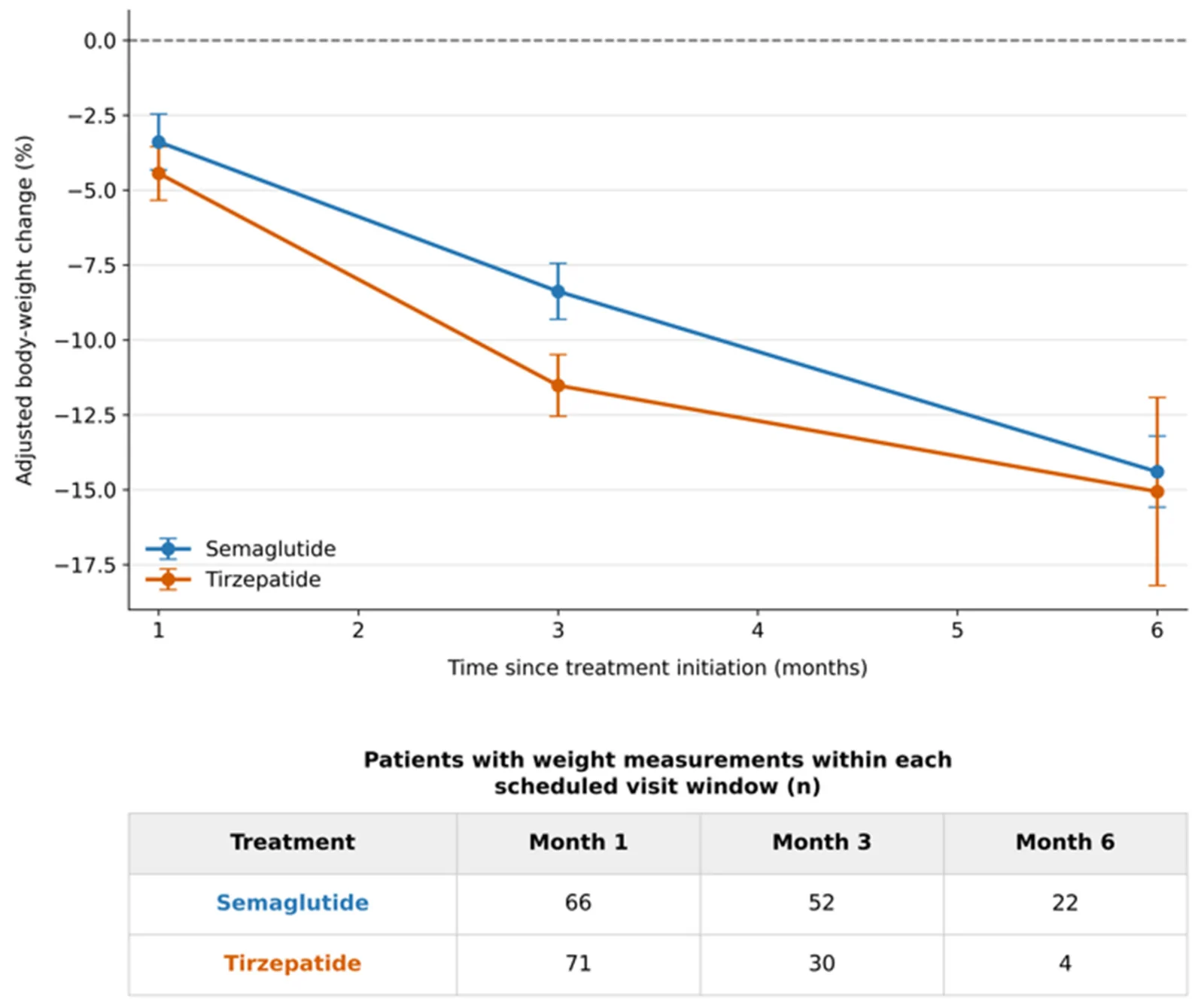
Model-adjusted percentage changes in body weight during semaglutide and tirzepatide treatment. Points represent model-adjusted mean percentage changes in body weight, error bars represent 95% confidence intervals, and lines connect the estimates at months 1, 3, and 6. Estimates were obtained from a linear mixed-effects model with a patient-specific random intercept and were adjusted for age, sex, baseline BMI, and diabetes status. Exact time since treatment initiation was modeled using linear and quadratic terms. Negative values indicate weight loss. The numbers shown below the graph represent the number of observations available at the scheduled month 1, month 3, and month 6 visits. Relatively few tirzepatide observations were available near month 6; therefore, the corresponding confidence interval was wider, and the estimate should be interpreted cautiously.

The primary mixed-effects analysis included 345 valid follow-up body-weight measurements obtained between days 1 and 230 from 148 patients, comprising 73 patients who initiated semaglutide and 75 who initiated tirzepatide. After adjustment for age, sex, baseline BMI, and diabetes status, the overall treatment-by-time interaction was statistically significant (Wald χ^2^[2] = 12.61, *p* = 0.002), indicating that the model-estimated weight-change trajectories differed between the treatment groups (Figure 2; Table 2). Multicollinearity was not evident among the prespecified baseline covariates. VIF values were 1.09 for the treatment group, 1.47 for age, 1.05 for sex, 1.05 for baseline BMI, and 1.44 for diabetes status; all values were below the prespecified threshold of 5.

At month 1, the model-adjusted mean percentage change in body weight was −3.39% (95% CI, −4.32 to −2.46) with semaglutide and −4.44% (95% CI, −5.34 to −3.55) with tirzepatide. The adjusted between-group difference, calculated as tirzepatide minus semaglutide, was −1.06 percentage points (95% CI, −2.38 to 0.26; *p* = 0.116) (Table 2).

At month 3, the model-adjusted mean percentage change was −8.38% (95% CI, −9.31 to −7.44) with semaglutide and −11.52% (95% CI, −12.55 to −10.49) with tirzepatide. The model-estimated weight reduction was 3.14 percentage points larger with tirzepatide than with semaglutide (between-group difference, −3.14 percentage points; 95% CI, −4.55 to −1.74; *p* < 0.001) (Figure 2; Table 2).

At month 6, the model-adjusted mean percentage changes were −14.40% (95% CI, −15.58 to −13.21) with semaglutide and −15.06% (95% CI, −18.20 to −11.92) with tirzepatide. The adjusted between-group difference was −0.66 percentage points (95% CI, −4.04 to 2.71; *p* = 0.699). The confidence interval around the tirzepatide estimate was wider at this time point because relatively few tirzepatide observations were available near month 6. This limited availability should be interpreted in the context of the later calendar-time initiation of tirzepatide and the substantially shorter potential follow-up duration in this group (Figure 2; Table 2).

In the sensitivity analysis treating follow-up time as a categorical variable, the overall treatment-by-time interaction remained statistically significant (Wald χ^2^[2] = 15.35, *p* < 0.001). The adjusted between-group differences, calculated as tirzepatide minus semaglutide, were −0.87 percentage points at month 1 (95% CI, −2.27 to 0.54; *p* = 0.226), −3.92 percentage points at month 3 (95% CI, −5.60 to −2.24; *p* < 0.001), and −1.39 percentage points at month 6 (95% CI, −4.81 to 2.03; *p* = 0.426). Thus, the categorical-time analysis was consistent with the primary quadratic-time model, showing larger model-estimated weight reduction with tirzepatide at month 3 but no evidence of a between-group difference at months 1 or 6 (Supplementary Table S3). The month 6 estimate remained imprecise because of the limited number of tirzepatide observations.

In the additional sensitivity analysis adjusting for calendar-time treatment initiation, the overall treatment-by-time interaction remained statistically significant (Wald χ^2^[2] = 13.39, *p* = 0.001). The adjusted between-group differences, calculated as tirzepatide minus semaglutide, were −0.75 percentage points at month 1 (95% CI, −2.27 to 0.77; *p* = 0.330), −2.94 percentage points at month 3 (95% CI, −4.54 to −1.34; *p* < 0.001), and −1.03 percentage points at month 6 (95% CI, −4.47 to 2.40; *p* = 0.555). Additional adjustment for treatment initiation date, therefore, did not materially alter the principal findings (Supplementary Table S4).

### 3.4. Anthropometric Changes at Scheduled Follow-Up Visits

In the supportive scheduled-visit analysis, paired baseline and month 1 measurements were available for 137 patients. Mean body weight decreased from 97.62 ± 18.44 to 93.55 ± 17.93 kg, corresponding to a mean change of −4.08 kg (95% CI, −4.58 to −3.57; *p* < 0.001; Cohen’s d^z^ = −1.36). Mean BMI decreased by 1.45 kg/m^2^ (95% CI, −1.62 to −1.28; *p* < 0.001), waist circumference by 3.48 cm (95% CI, −4.05 to −2.91; *p* < 0.001), and hip circumference by 2.82 cm (95% CI, −3.21 to −2.44; *p* < 0.001). The waist-to-hip ratio also decreased, with a mean change of −0.009 (95% CI, −0.013 to −0.004; *p* < 0.001) (Table 3).

At month 3, paired anthropometric measurements were available for 82 patients. Mean body weight decreased from 97.37 ± 18.72 to 87.91 ± 17.87 kg, corresponding to a mean change of −9.45 kg (95% CI, −10.69 to −8.22; *p* < 0.001; Cohen’s d^z^ = −1.69). Mean BMI decreased by 3.36 kg/m^2^ (95% CI, −3.77 to −2.96; *p* < 0.001), waist circumference by 7.15 cm (95% CI, −8.18 to −6.11; *p* < 0.001), and hip circumference by 7.12 cm (95% CI, −8.17 to −6.08; *p* < 0.001). The waist-to-hip ratio decreased by 0.012 (95% CI, −0.021 to −0.002; *p* = 0.016) (Table 3).

At month 6, paired measurements were available for 26 patients. Mean body weight decreased from 99.80 ± 22.17 to 86.43 ± 19.23 kg, corresponding to a mean change of −13.37 kg (95% CI, −16.65 to −10.08; *p* < 0.001; Cohen’s d^z^ = −1.64). Mean BMI decreased by 4.66 kg/m^2^ (95% CI, −5.65 to −3.68; *p* < 0.001), waist circumference by 10.88 cm (95% CI, −13.56 to −8.21; *p* < 0.001), and hip circumference by 9.85 cm (95% CI, −11.92 to −7.77; *p* < 0.001). A smaller reduction was observed in waist-to-hip ratio (mean change, −0.020; 95% CI, −0.039 to −0.001; *p* = 0.042) (Table 3). Because different patient subsets contributed to each scheduled visit, these unadjusted changes were not directly compared across time points.

### 3.5. Clinically Relevant Weight-Loss Responses

At month 1, 54 of 137 patients (39.4%) achieved at least 5% weight loss. This threshold was achieved by 47.9% of tirzepatide-treated patients and 30.3% of semaglutide-treated patients (*p* = 0.035). Only two patients (1.5%), both in the semaglutide group, achieved at least 10% weight loss. No patient achieved the 15% or 20% thresholds at month 1 (Table 4).

At month 3, 67 of 82 patients (81.7%) achieved at least 5% weight loss, with corresponding proportions of 90.0% in the tirzepatide group and 76.9% in the semaglutide group (*p* = 0.140). At least 10% weight loss was achieved by 48.8% of patients overall and was more frequent with tirzepatide than with semaglutide (63.3% vs. 40.4%; *p* = 0.045). Similarly, at least 15% weight loss was achieved by 26.7% of tirzepatide-treated patients and 5.8% of semaglutide-treated patients (*p* = 0.015). The proportion achieving at least 20% weight loss was low and did not differ between the groups (3.3% vs. 1.9%; *p* = 1.000) (Table 4).

Among the 26 patients with month 6 measurements, 24 (92.3%) achieved at least 5% weight loss, 18 (69.2%) achieved at least 10%, 11 (42.3%) achieved at least 15%, and 4 (15.4%) achieved at least 20% weight loss. No statistically significant between-group differences were observed for any month 6 response threshold (all *p* > 0.05). However, these unadjusted comparisons should be interpreted cautiously because only four tirzepatide-treated patients contributed to the scheduled month 6 analysis (Table 4).

### 3.6. Metabolic and Visceral Adiposity Outcomes

Paired month 3 analyses showed nominally significant changes in several glycemic and metabolic variables. Median HbA1c decreased from 5.75% (5.50–6.10) at baseline to 5.40% (5.20–5.78) at month 3, with a median within-participant change of −0.30 percentage points (−0.58 to −0.13; nominal *p* < 0.001). Median fasting glucose decreased by 7.9 mg/dL (−13.0 to −4.2; nominal *p* < 0.001), fasting insulin by 4.40 µIU/mL (−8.90 to 0.09; nominal *p* < 0.001), and HOMA-IR by 1.40 (−3.01 to −0.24; nominal *p* < 0.001) (Table 5).

Among the lipid-related outcomes, median LDL cholesterol decreased by 11.0 mg/dL (−31.5 to −3.2; nominal *p* < 0.001), and triglycerides decreased by 27.0 mg/dL (−47.0 to −0.5; nominal *p* = 0.001). HDL cholesterol did not show evidence of a change (median change, −1.0 mg/dL; −4.2 to 2.0; *p* = 0.270). The TyG index decreased by 0.312 (−0.491 to −0.104; nominal *p* < 0.001). AIP showed a more modest change of −0.084 (−0.163 to 0.017; nominal *p* = 0.042) (Table 5).

The evaluated surrogate indices of visceral adiposity also showed nominal reductions at month 3. Median LAP decreased by 20.24 units (−35.32 to −5.39; nominal *p* < 0.001), whereas VAI showed a modest change of −0.38 (−0.77 to 0.01; nominal *p* = 0.030). TyG-BMI decreased by 40.46 units (−59.38 to −26.80; nominal *p* < 0.001), and TyG–waist circumference decreased by 94.90 units (−155.71 to −63.63; nominal *p* < 0.001) (Table 5). Because these secondary analyses were not adjusted for multiple comparisons, their *p* values should be interpreted as exploratory.

### 3.7. Hepatic and Inflammatory Outcomes

At month 3, median ALT decreased by 4.5 U/L (−10.5 to 3.5; nominal *p* = 0.020), AST by 3.0 U/L (−5.0 to 0.0; nominal *p* = 0.005), and GGT by 4.5 U/L (−6.0 to 0.0; nominal *p* < 0.001) (Table 5).

There was no evidence of a change in CRP. Median CRP was 3.50 mg/L (1.60–6.00) at baseline and 2.10 mg/L (1.60–5.55) at month 3, corresponding to a median within-participant change of −0.40 mg/L (−1.15 to 0.70; *p* = 0.461) (Table 5).

Exploratory analyses based on seven paired observations showed decreases in total cholesterol and non-HDL cholesterol, with median changes of −24.0 and −18.2 mg/dL, respectively (both nominal *p* = 0.016). ESR, assessed in only four patients, did not show evidence of a change (median change, 8.5 mm/h; *p* = 0.500). Given the very small numbers of paired observations, these findings were considered descriptive and hypothesis-generating (Supplementary Table S2).

## 4. Discussion

In this real-world longitudinal cohort, both semaglutide and tirzepatide were associated with substantial early reductions in body weight. The model-estimated trajectories differed significantly between the treatment groups, with tirzepatide being associated with a larger model-adjusted reduction at month 3, whereas no statistically significant between-group difference was observed at month 1. The month 3 finding remained consistent when follow-up time was modeled categorically and after additional adjustment for calendar-time treatment initiation, supporting the robustness of the principal early comparative result. Reductions in body weight were accompanied by improvements in BMI, waist and hip circumferences, and several glycemic, lipid, hepatic, and surrogate visceral adiposity measures.

Interpreting the baseline characteristics and follow-up outcomes together provides important context for the comparative findings. The cohort was predominantly female (75.5%), with a mean age of 41.7 years and BMI of 35.0 kg/m^2^; only 12.5% had type 2 diabetes. The findings, therefore, primarily describe a relatively young, predominantly female population receiving treatment for obesity. Baseline anthropometric measures were numerically similar between groups, although nonsignificant baseline comparisons do not establish equivalence or exclude confounding. The proportion with diabetes was numerically higher with semaglutide (16.0% versus 8.9%), supporting its inclusion among the adjustment variables. More importantly, tirzepatide was initiated later and had shorter potential follow-up, which helps explain the sparse month 6 observations and limits interpretation of the apparent proximity of the later model-estimated trajectories.

The continuous and categorical weight outcomes were broadly consistent at month 3: the adjusted reduction was 3.14 percentage points larger with tirzepatide (95% CI, 1.74–4.55; *p* < 0.001), and 63.3% versus 40.4% achieved at least 10% weight loss (nominal *p* = 0.045). At month 1, however, the unadjusted ≥5% response proportions differed, while the adjusted mean contrast was not statistically significant, illustrating that threshold-based responses and adjusted continuous outcomes address different aspects of the weight-loss distribution. The pooled month 3 reductions in weight (−9.45 kg) and waist circumference (−7.15 cm) were accompanied by reductions in HOMA-IR, triglycerides, and TyG, consistent with concurrent anthropometric and metabolic changes. Nevertheless, the laboratory subsets differed in size, the analyses were exploratory, and changes in composite indices partly reflect changes in their mathematical components. These findings neither establish individual-level correlations between weight loss and metabolic improvement nor demonstrate the metabolic superiority of either treatment.

The larger model-estimated weight reduction with tirzepatide at month 3 is consistent with accumulating comparative evidence suggesting that a treatment difference may emerge relatively early during dose escalation. In a recent propensity-score-weighted US electronic health record study, tirzepatide was associated with a larger reduction in body weight than semaglutide at 6 months, together with higher probabilities of achieving clinically relevant weight-loss thresholds [19]. Earlier randomized evidence in patients with type 2 diabetes similarly demonstrated larger reductions in body weight with tirzepatide than with semaglutide 1 mg across all evaluated tirzepatide doses [20]. A 2025 systematic review and network meta-analysis of 56 randomized trials also identified substantial weight-loss efficacy for semaglutide and tirzepatide [21]. This evidence provides broader context for our early weight-loss findings, although the longer treatment durations and protocol-defined doses limit direct comparison of effect magnitudes with those observed in our short-term routine-care cohort. The present study extends these observations to a routine-care cohort from Türkiye that included patients with and without diabetes and used exact follow-up time rather than restricting the primary analysis to fixed visit windows. Nevertheless, because treatment allocation was not randomized, the observed difference should be interpreted as an adjusted association rather than proof of comparative causal effectiveness.

A recent Turkish single-center study of 169 adults without diabetes reported larger weight loss with tirzepatide than with semaglutide and liraglutide at 36 weeks [11]. Compared with that study, the present study included patients with and without type 2 diabetes and evaluated all available repeated body-weight measurements using exact follow-up time. In addition to characterizing early longitudinal body-weight trajectories, we evaluated changes in hip circumference, waist-to-hip ratio, inflammatory markers, and derived metabolic indices, including the TyG index and AIP, which were not comprehensively assessed in the previous Turkish comparative cohort. Our findings therefore complement the previous Turkish experience by describing early weight trajectories and related anthropometric and metabolic changes under irregular and unequal follow-up conditions.

At month 1, the adjusted between-group difference was modest and did not reach statistical significance, whereas a clearer separation was evident at month 3. Both medications were initiated at low doses and progressively escalated in their pivotal obesity trials [5,6]. Early responses in the present cohort may therefore reflect initial exposure rather than the full treatment effect. By month 6, the model-estimated reductions were −14.40% with semaglutide and −15.06% with tirzepatide, but the between-group contrast was imprecise and not statistically significant. This result should not be interpreted as evidence that the effects of the treatments converged. The month 6 tirzepatide estimate was primarily model-based and should be considered exploratory because of sparse observations near this time point. Accordingly, the month 3 estimate represents the most reliable comparative finding of the present study, whereas longer-term comparative effectiveness requires cohorts with more balanced follow-up opportunity.

The scheduled-visit analyses further demonstrated that the observed weight reductions were clinically meaningful at the individual-patient level. By month 3, more than four-fifths of patients had achieved at least 5% weight loss, nearly half had achieved at least 10%, and the proportions achieving at least 10% and 15% weight loss were higher among tirzepatide-treated patients. These categorical outcomes complement the mean trajectory estimates by showing that the treatment-associated changes were not driven solely by a small number of marked responders. Real-world studies have likewise reported clinically meaningful 6-month weight-loss responses with semaglutide and tirzepatide, although the magnitude of weight loss has varied according to achieved dose, treatment persistence, population characteristics, and the availability of follow-up measurements [22,23]. The high response proportions observed at month 6 in the present cohort should nevertheless be interpreted cautiously because they were calculated among the limited subset with available paired measurements, including only four tirzepatide-treated patients.

The reductions in waist and hip circumferences provide complementary information beyond changes in body weight and BMI. Waist circumference decreased by approximately 3.5 cm at month 1, 7.2 cm at month 3, and 10.9 cm at month 6 among patients with paired measurements. Waist circumference provides information on abdominal adiposity and cardiometabolic risk that is not fully captured by BMI, and international consensus recommendations have emphasized its value both for risk assessment and as a treatment target in patients with overweight or obesity [24]. Although the different patient subsets contributing to each visit preclude direct comparisons across time points, the parallel reductions in weight, BMI, and waist circumference support a broad improvement in anthropometric risk profiles during treatment.

Exploratory month 3 analyses also showed improvements in HbA1c, fasting glucose, fasting insulin, HOMA-IR, LDL cholesterol, triglycerides, and the TyG index. These findings are compatible with the known metabolic effects of incretin-based therapies and with real-world observations showing that semaglutide- or tirzepatide-associated weight reduction may be accompanied by improvements in glycemic and cardiometabolic parameters [19,25]. Exploratory analyses of STEP 1 and STEP 4 provide more specific randomized-trial context for these observations. In STEP 1, semaglutide produced larger reductions than placebo in waist circumference, fasting glucose, fasting insulin, HOMA-IR, and lipid measures [26]. The direction of these changes is consistent with our pooled metabolic findings, although differences in treatment duration, dosing, diabetes eligibility, and laboratory assessment prevent direct comparison of their magnitude. Our uncontrolled within-participant analyses also cannot distinguish medication effects from concurrent weight loss or other changes in care. However, the present metabolic analyses pooled the available paired observations across treatment groups and were designed to characterize within-participant changes rather than estimate comparative treatment effects. They should therefore not be used to conclude that one agent produced more pronounced metabolic improvement than the other.

Reductions were also observed in LAP, VAI, TyG-BMI, and TyG–waist circumference. These indices combine anthropometric and biochemical measurements. LAP was developed as an indicator of lipid overaccumulation associated with cardiovascular risk, whereas VAI was developed to provide an indirect estimate of visceral adipose dysfunction and cardiometabolic risk [17,18]. Their direction of change was consistent with the simultaneous reductions in waist circumference, BMI, fasting glucose, and triglycerides. Nevertheless, these measures are calculated surrogates rather than direct assessments of visceral fat or organ-level metabolic improvement. Because several components of these formulas also constituted observed outcomes, their changes are partly mathematically related to the accompanying anthropometric and lipid reductions. The findings should therefore be viewed as supportive and hypothesis-generating rather than as independent evidence of reduced visceral adipose tissue.

The modest reductions in ALT, AST, and GGT observed at month 3 are also biologically consistent with potential improvement in obesity-associated hepatic metabolic stress. Randomized trials have demonstrated favorable hepatic effects of incretin-based therapies in selected patients with biopsy-confirmed metabolic dysfunction-associated steatohepatitis. In the phase 3 ESSENCE trial, semaglutide improved steatohepatitis resolution and fibrosis-related histologic outcomes compared with placebo [27], while the phase 2 SYNERGY-NASH trial demonstrated higher rates of steatohepatitis resolution with tirzepatide in patients with moderate or severe fibrosis [28]. However, the present study did not systematically evaluate hepatic steatosis, fibrosis, imaging findings, or histology. Consequently, the observed enzyme reductions cannot establish improvement in metabolic dysfunction-associated steatotic liver disease (MASLD) or metabolic dysfunction-associated steatohepatitis (MASH) and should be interpreted only as preliminary biochemical findings.

In contrast to the other metabolic markers, CRP did not change significantly at month 3. This differs from analyses of longer-duration semaglutide trials in which reductions in CRP were observed after treatment and were correlated with changes in body weight, waist circumference, glucose, insulin, and HOMA-IR [29]. The absence of a detectable CRP change in the present cohort may reflect the relatively short assessment period, modest baseline inflammatory burden, biological variability, and limited availability of paired measurements rather than the absence of an anti-inflammatory effect. It also illustrates why metabolic and inflammatory outcomes obtained during routine care should be interpreted separately from the more comprehensively measured body-weight outcome.

Overall, these findings add region-specific real-world evidence showing that clinically meaningful weight reduction with semaglutide and tirzepatide can occur during the first months of routine treatment. The use of all available repeated body-weight measurements and exact follow-up time enabled the analysis to accommodate irregular visit timing, while the categorical-time- and calendar-time-adjusted sensitivity analyses supported the principal month 3 finding. At the same time, the results emphasize the importance of distinguishing directly observed scheduled-visit outcomes from model-derived estimates when follow-up opportunity differs between treatment groups. This distinction is particularly relevant in rapidly changing clinical settings in which newer therapies enter practice later and consequently have fewer patients available for longer-term evaluation.

## 5. Limitations

This study has several limitations. First, its retrospective, single-center, observational design precludes causal inference and may limit the generalizability of the findings to other clinical settings and populations. Treatment selection was determined by routine clinical practice rather than randomization; therefore, residual confounding related to clinical characteristics, patient preferences, medication access, dosing, adherence, and treatment persistence may remain despite adjustment for age, sex, baseline BMI, diabetes status, and calendar-time treatment initiation.

No treatment switching was documented during follow-up; however, detailed data on dose escalation, temporary interruptions, treatment persistence, and discontinuation were not systematically available. Therefore, the findings should be interpreted as initiation-based rather than dose-specific or strictly on-treatment effectiveness estimates.

Second, treatment initiation occurred later in the tirzepatide group, resulting in substantially fewer patients with the opportunity to reach 6 months of follow-up by the data cut-off date. Consequently, the month 6 tirzepatide estimate was based on limited data and was considerably less precise. The small number of later observations reduced statistical power to detect between-group differences and widened confidence intervals. Patients contributing later measurements may also differ from the baseline cohort, limiting the representativeness of the later findings. Using all available measurements in the mixed-effects model does not eliminate potential bias from informative missingness. Although the mixed-effects model incorporated exact follow-up time and all available repeated measurements, residual calendar-time and follow-up-availability bias cannot be excluded, particularly for longer-term between-treatment comparisons. The month 6 tirzepatide estimate should therefore be considered exploratory and should not be interpreted as evidence of equivalence between the treatments.

Third, follow-up assessments were performed as part of routine clinical care and were not scheduled uniformly. Furthermore, 36 of the 184 eligible patients had no valid follow-up body-weight measurement and were excluded from the longitudinal analysis. Because patients with and without available follow-up measurements were not formally compared, selection bias related to follow-up availability cannot be excluded. The mixed-effects model uses available observations under a missing-at-random assumption conditional on the included variables; however, this assumption cannot be verified, and informative missingness remains possible. Similarly, scheduled-visit analyses were based on available paired measurements and involved different patient subsets at each time point, preventing direct comparison of the unadjusted changes across visits.

Fourth, metabolic and laboratory outcomes were available only in relatively small subsets of patients who underwent repeat testing during routine clinical care. Because repeat laboratory assessment was not standardized and may have been influenced by clinical characteristics, these analyses are vulnerable to selection and measurement-availability bias. Moreover, the metabolic analyses evaluated within-participant changes rather than comparative treatment effects and were not adjusted for multiple testing. These findings should therefore be considered exploratory and hypothesis-generating.

Fifth, the derived adiposity indices represent calculated surrogate outcomes rather than direct measurements of visceral adipose tissue. Because these indices incorporate BMI, waist circumference, and lipid or glucose measurements, their changes are mathematically dependent on changes in these components. Consequently, reductions in several indices should not be interpreted as multiple independent confirmations of visceral fat reduction. Their use provides descriptive information on changes in combined anthropometric and biochemical profiles, but the absence of imaging prevents confirmation of corresponding changes in visceral fat volume. Similarly, reductions in liver enzymes represent biochemical changes and cannot establish improvement in hepatic steatosis, steatohepatitis, or fibrosis without imaging, elastography, or histologic assessment. These methodological limitations constrain the biological interpretation of the exploratory secondary outcomes; they do not directly affect the calculation of the primary body-weight outcome or resolve the separate confounding and missing-data limitations of its analysis.

Finally, the study primarily evaluated the early treatment period. The available data did not permit robust assessment of longer-term weight-loss durability, weight regain, treatment discontinuation, longer-term switching patterns, or safety outcomes. Larger multicenter studies with standardized follow-up, protocolized dose-escalation schedules, balanced calendar-time exposure, and longer observation periods are needed to confirm the comparative trajectories identified in this cohort.

## 6. Conclusions

This study provides comparative evidence on early weight-loss trajectories following semaglutide and tirzepatide initiation in routine care in Türkiye among adults with obesity or overweight with weight-related comorbidities, with and without type 2 diabetes. Both treatments were associated with early reductions in body weight. Addressing the primary comparative objective, tirzepatide was associated with larger model-adjusted weight loss at month 3, whereas no statistically significant between-group difference was observed at month 1. By incorporating irregularly timed measurements using exact follow-up time, the study adds evidence on early treatment trajectories in a clinical population that differs from protocol-defined randomized trial populations.

Among participants with paired baseline and month 3 measurements, reductions in body weight and waist circumference were accompanied by improvements in several glycemic, insulin-resistance, and lipid measures. These observations suggest potentially favorable changes in adiposity and metabolic risk markers during early treatment. However, the pooled metabolic analyses do not establish comparative metabolic efficacy, effects independent of weight loss, or reductions in obesity-related complications.

Nonrandomized treatment allocation, the modest sample size, incomplete follow-up, and the selected study population limit causal interpretation and generalizability. Sparse month 6 observations in the tirzepatide group preclude a reliable longer-term comparison. These findings may help contextualize early weight-loss responses in routine care, but are insufficient to establish treatment superiority or guide treatment selection on their own. Larger prospective comparative studies with balanced follow-up, standardized metabolic assessments, and documentation of dose escalation and treatment persistence are needed to evaluate sustained weight loss and longer-term clinical outcomes.

## Figures and Tables

**Table 1:** Baseline demographic and clinical characteristics and follow-up opportunity according to treatment group.

Variable	Overall (*n* = 184)	Semaglutide (*n* = 94)	Tirzepatide (*n* = 90)	Test Statistic	*p*
Age, years	41.7 ± 12.0	40.4 ± 12.1	43.1 ± 11.7	t = −1.53	0.127
Height, cm	166.2 ± 8.5	166.6 ± 9.4	165.8 ± 7.6	t = 0.60	0.551
Body weight, kg	97.0 ± 17.9	96.3 ± 18.5	97.7 ± 17.3	t = −0.51	0.611
BMI, kg/m^2^	35.00 ± 5.30	34.63 ± 5.66	35.38 ± 4.90	t = −0.96	0.336
Waist circumference, cm	105.6 ± 13.9	105.3 ± 14.0	105.9 ± 13.9	t = −0.27	0.786
Hip circumference, cm	121.3 ± 11.5	121.0 ± 12.7	121.7 ± 10.3	t = −0.38	0.704
Waist-to-hip ratio	0.871 ± 0.090	0.872 ± 0.094	0.870 ± 0.086	t = 0.18	0.861
Female sex, *n* (%)	139 (75.5)	70 (74.5)	69 (76.7)	χ^2^ = 0.12	0.729
Type 2 diabetes mellitus, *n* (%)	23 (12.5)	15 (16.0)	8 (8.9)	χ^2^ = 2.10	0.147
Hypertension, *n* (%)	17 (9.2)	6 (6.4)	11 (12.2)	χ^2^ = 1.87	0.172
Hyperlipidemia, *n* (%)	8 (4.3)	6 (6.4)	2 (2.2)	Fisher’s exact test	0.279
Atherosclerotic cardiovascular disease, *n* (%)	3 (1.6)	3 (3.2)	0 (0.0)	Fisher’s exact test	0.246
**Treatment timing and follow-up opportunity**					
Treatment initiation date	3 November 2025 (19 September–15 December 2025)	22 September 2025 (27 July–3 December 2025)	24 November 2025 (19 October–30 December 2025)	U = 2562.0	<0.001
Potential follow-up duration at data cut-off, days	129 (86–171)	169 (98–225)	108 (71–141)	U = 2562.0	<0.001
Eligible to enter the month 6 follow-up window, *n*/*N* (%)	66/184 (35.9)	52/94 (55.3)	14/90 (15.6)	χ^2^ = 31.60	<0.001

Data are presented as mean ± standard deviation, median (Q1–Q3), or number (percentage), as appropriate. Continuous variables were compared using Welch’s independent-samples *t*-test or the Mann–Whitney U test, as appropriate. Categorical variables were compared using Pearson’s chi-square test or Fisher’s exact test, as appropriate. Treatment initiation dates and potential follow-up durations were available for all 184 patients. Potential follow-up duration was calculated from the treatment initiation date to the data cut-off date of 10 March 2026. Patients with at least 150 days between treatment initiation and the data cut-off date were considered eligible to enter the month 6 follow-up window. BMI, body mass index.

**Table 2:** Model-adjusted percentage changes in body weight according to treatment group.

Follow-Up	Semaglutide, Adjusted % Change (95% CI)	Tirzepatide, Adjusted % Change (95% CI)	Adjusted Between-Group Difference (95% CI)	z	*p*
Month 1	−3.39 (−4.32 to −2.46)	−4.44 (−5.34 to −3.55)	−1.06 (−2.38 to 0.26)	−1.57	0.116
Month 3	−8.38 (−9.31 to −7.44)	−11.52 (−12.55 to −10.49)	−3.14 (−4.55 to −1.74)	−4.38	<0.001
Month 6	−14.40 (−15.58 to −13.21)	−15.06 (−18.20 to −11.92)	−0.66 (−4.04 to 2.71)	−0.39	0.699

Values are model-adjusted mean percentage changes in body weight with 95% confidence intervals. The between-group difference was calculated as tirzepatide minus semaglutide; therefore, negative values indicate a larger model-estimated weight reduction with tirzepatide. Estimates were obtained using a linear mixed-effects model with a patient-specific random intercept. Exact time elapsed since treatment initiation was modeled using linear and quadratic time terms. The model included treatment group, time, treatment-by-time interactions, age, sex, baseline BMI, and diabetes status. The analysis included 345 follow-up measurements from 148 patients: 73 initially treated with semaglutide and 75 initially treated with tirzepatide. Follow-up measurements obtained from day 1 through day 230 were included. The overall treatment-by-time interaction was statistically significant, χ^2^(2) = 12.61, *p* = 0.002. Because relatively few tirzepatide observations were available near month 6, the month 6 tirzepatide estimate was less precise and should be interpreted cautiously. Treatment groups were defined according to the treatment initiated at baseline. These observational estimates should not be interpreted as causal comparative-effectiveness effects. BMI, body mass index; CI, confidence interval. At the scheduled follow-up visits, the numbers of observations in the semaglutide and tirzepatide groups were 66 and 71 at month 1, 52 and 30 at month 3, and 22 and 4 at month 6, respectively. The primary mixed-effects model additionally incorporated valid intermediate measurements obtained between days 1 and 230.

**Table 3:** Changes in anthropometric measures at scheduled follow-up visits.

Follow-Up	Outcome	*n*	Baseline	Follow-Up	Mean Change (95% CI)	t	*p*	Cohen’s d^z^
Month 1	Body weight, kg	137	97.62 ± 18.44	93.55 ± 17.93	−4.08 (−4.58 to −3.57)	−15.89	<0.001	−1.36
Month 1	BMI, kg/m^2^	137	34.91 ± 5.25	33.46 ± 5.22	−1.45 (−1.62 to −1.28)	−16.82	<0.001	−1.44
Month 1	Waist circumference, cm	137	105.66 ± 14.63	102.18 ± 14.46	−3.48 (−4.05 to −2.91)	−12.14	<0.001	−1.04
Month 1	Hip circumference, cm	137	121.64 ± 11.04	118.81 ± 10.89	−2.82 (−3.21 to −2.44)	−14.58	<0.001	−1.25
Month 1	Waist-to-hip ratio	137	0.869 ± 0.092	0.860 ± 0.092	−0.009 (−0.013 to −0.004)	−4.03	<0.001	−0.34
Month 3	Body weight, kg	82	97.37 ± 18.72	87.91 ± 17.87	−9.45 (−10.69 to −8.22)	−15.27	<0.001	−1.69
Month 3	BMI, kg/m^2^	82	34.67 ± 5.38	31.31 ± 5.38	−3.36 (−3.77 to −2.96)	−16.51	<0.001	−1.82
Month 3	Waist circumference, cm	82	105.26 ± 14.84	98.11 ± 14.58	−7.15 (−8.18 to −6.11)	−13.70	<0.001	−1.51
Month 3	Hip circumference, cm	82	121.40 ± 11.61	114.28 ± 12.08	−7.12 (−8.17 to −6.08)	−13.57	<0.001	−1.50
Month 3	Waist-to-hip ratio	82	0.867 ± 0.090	0.855 ± 0.086	−0.012 (−0.021 to −0.002)	−2.46	0.016	−0.27
Month 6	Body weight, kg	26	99.80 ± 22.17	86.43 ± 19.23	−13.37 (−16.65 to −10.08)	−8.37	<0.001	−1.64
Month 6	BMI, kg/m^2^	26	35.19 ± 6.04	30.53 ± 5.76	−4.66 (−5.65 to −3.68)	−9.79	<0.001	−1.92
Month 6	Waist circumference, cm	26	110.12 ± 16.96	99.23 ± 17.10	−10.88 (−13.56 to −8.21)	−8.39	<0.001	−1.64
Month 6	Hip circumference, cm	26	122.35 ± 12.31	112.50 ± 11.81	−9.85 (−11.92 to −7.77)	−9.78	<0.001	−1.92
Month 6	Waist-to-hip ratio	26	0.898 ± 0.088	0.878 ± 0.088	−0.020 (−0.039 to −0.001)	−2.14	0.042	−0.42

Data are presented as mean ± standard deviation unless otherwise indicated. The mean change was calculated as the follow-up value minus the baseline value. Each follow-up time point was analyzed separately among participants with both baseline and corresponding follow-up measurements using the paired-samples *t*-test. The 95% confidence intervals refer to the mean within-participant changes. Cohen’s d^z^ represents the standardized effect size for paired measurements. Because the number and composition of participants differed across follow-up visits, unadjusted changes should not be directly compared between time points. BMI, body mass index; CI, confidence interval.

**Table 4:** Proportions of patients achieving clinically relevant weight-loss thresholds at scheduled follow-up visits.

Follow-Up	Weight-Loss Threshold	Overall, *n* (%)	Semaglutide, *n* (%)	Tirzepatide, *n* (%)	Test Statistic	*p*
Month 1	≥5%	54 (39.4)	20 (30.3)	34 (47.9)	χ^2^ = 4.43	0.035
Month 1	≥10%	2 (1.5)	2 (3.0)	0 (0.0)	Fisher’s exact test	0.230
Month 1	≥15%	0 (0.0)	0 (0.0)	0 (0.0)	—	—
Month 1	≥20%	0 (0.0)	0 (0.0)	0 (0.0)	—	—
Month 3	≥5%	67 (81.7)	40 (76.9)	27 (90.0)	χ^2^ = 2.18	0.140
Month 3	≥10%	40 (48.8)	21 (40.4)	19 (63.3)	χ^2^ = 4.01	0.045
Month 3	≥15%	11 (13.4)	3 (5.8)	8 (26.7)	Fisher’s exact test	0.015
Month 3	≥20%	2 (2.4)	1 (1.9)	1 (3.3)	Fisher’s exact test	1.000
Month 6	≥5%	24 (92.3)	21 (95.5)	3 (75.0)	Fisher’s exact test	0.289
Month 6	≥10%	18 (69.2)	15 (68.2)	3 (75.0)	Fisher’s exact test	1.000
Month 6	≥15%	11 (42.3)	10 (45.5)	1 (25.0)	Fisher’s exact test	0.614
Month 6	≥20%	4 (15.4)	3 (13.6)	1 (25.0)	Fisher’s exact test	0.511

Data are presented as number (percentage). Percentages were calculated using the number of participants with available body-weight measurements at each follow-up visit as the denominator. The corresponding denominators were: month 1, overall *n* = 137, semaglutide *n* = 66, and tirzepatide *n* = 71; month 3, overall *n* = 82, semaglutide *n* = 52, and tirzepatide *n* = 30; and month 6, overall *n* = 26, semaglutide *n* = 22, and tirzepatide *n* = 4. Treatment groups were compared using Pearson’s chi-square test or Fisher’s exact test, as appropriate. A statistical comparison was not performed when no participant in either treatment group achieved the specified threshold. The comparisons were unadjusted for baseline characteristics and multiple testing. Owing to the observational design and marked imbalance in treatment-group availability at month 6, these findings should not be interpreted as causal comparative effectiveness estimates.

**Table 5:** Changes in metabolic, visceral adiposity, hepatic, and inflammatory markers at month 3.

Variable	*n*	Baseline	Month 3	Change	W	*p*
HbA1c, %	34	5.75 (5.50–6.10)	5.40 (5.20–5.78)	−0.30 (−0.58 to −0.13)	21.5	<0.001
Fasting glucose, mg/dL	47	98.8 (95.0–108.0)	92.1 (87.7–99.3)	−7.9 (−13.0 to −4.2)	113.0	<0.001
Fasting insulin, µIU/mL	44	16.25 (11.47–25.62)	12.80 (9.24–16.92)	−4.40 (−8.90 to 0.09)	154.5	<0.001
HOMA-IR	44	4.47 (2.66–8.16)	2.87 (1.97–3.94)	−1.40 (−3.01 to −0.24)	99.0	<0.001
LDL cholesterol, mg/dL	42	147.5 (130.0–165.8)	130.5 (118.0–157.8)	−11.0 (−31.5 to −3.2)	172.0	<0.001
HDL cholesterol, mg/dL	24	49.5 (40.2–58.2)	47.0 (40.0–54.5)	−1.0 (−4.2 to 2.0)	111.5	0.270
Triglycerides, mg/dL	40	125.5 (92.5–146.0)	88.5 (68.5–123.0)	−27.0 (−47.0 to −0.5)	171.0	0.001
TyG index	38	8.727 (8.374–8.917)	8.244 (8.057–8.656)	−0.312 (−0.491 to −0.104)	98.0	<0.001
AIP	23	0.043 (−0.171–0.187)	−0.070 (−0.263–0.077)	−0.084 (−0.163 to 0.017)	71.0	0.042
LAP	40	60.48 (36.94–94.33)	34.24 (22.37–62.66)	−20.24 (−35.32 to −5.39)	115.0	<0.001
VAI	23	1.70 (1.35–2.94)	1.32 (0.92–2.20)	−0.38 (−0.77 to 0.01)	67.0	0.030
TyG-BMI	38	293.17 (253.66–330.02)	242.28 (225.91–289.13)	−40.46 (−59.38 to −26.80)	0.0	<0.001
TyG–waist circumference	38	919.10 (771.80–1046.38)	773.21 (685.47–971.18)	−94.90 (−155.71 to −63.63)	18.0	<0.001
ALT, U/L	34	24.0 (15.2–33.8)	19.0 (14.2–30.8)	−4.5 (−10.5 to 3.5)	162.0	0.020
AST, U/L	29	20.0 (16.0–28.0)	18.0 (15.0–22.0)	−3.0 (−5.0 to 0.0)	64.0	0.005
GGT, U/L	24	14.0 (11.0–18.2)	11.0 (8.8–15.8)	−4.5 (−6.0 to 0.0)	19.0	<0.001
CRP, mg/L	27	3.50 (1.60–6.00)	2.10 (1.60–5.55)	−0.40 (−1.15 to 0.70)	146.5	0.461

Data are presented as median (Q1–Q3). Change was calculated as the month 3 value minus the baseline value and is also presented as a median (Q1–Q3), not as a confidence interval. Baseline and month 3 values were compared using the Wilcoxon signed-rank test; W denotes the Wilcoxon test statistic. HOMA-IR was calculated as fasting insulin × fasting glucose/405. The TyG index was calculated as ln[triglycerides × fasting glucose/2]. AIP was calculated as log10[triglycerides (mmol/L)/HDL cholesterol (mmol/L)]. LAP and VAI were calculated using sex-specific formulas. Laboratory analyses were restricted to participants with paired baseline and month 3 measurements; therefore, the sample size varied across outcomes. No adjustment for multiple comparisons was applied; consequently, secondary and exploratory findings should be interpreted as hypothesis-generating. HOMA-IR, homeostatic model assessment of insulin resistance; LDL, low-density lipoprotein; HDL, high-density lipoprotein; TyG, triglyceride-glucose index; AIP, atherogenic index of plasma; LAP, lipid accumulation product; VAI, visceral adiposity index; BMI, body mass index; ALT, alanine aminotransferase; AST, aspartate aminotransferase; GGT, gamma-glutamyl transferase; CRP, C-reactive protein.

## Data Availability

The data underlying this study are available from the corresponding author upon reasonable request, subject to ethical and institutional restrictions.

## Supplementary Materials

The following supporting information can be downloaded at https://www.mdpi.com/article/10.3390/jcm15207876/s1; Table S1. Baseline laboratory and derived metabolic characteristics according to treatment group. Table S2. Exploratory changes in laboratory outcomes with limited paired observations at month 3. Table S3. Sensitivity analysis of model-adjusted percentage body-weight change using categorical follow-up time. Table S4. Sensitivity analysis of model-adjusted percentage body-weight change with additional adjustment for calendar-time treatment initiation.
