# Supplementary material for: Body Weight Changes and Exploratory Metabolic Outcomes During Semaglutide and Tirzepatide Treatment: A Real-World Longitudinal Cohort Study from Türkiye
Source: J Clin Med. 2026 Oct 13;15(20):7876. doi: 10.3390/jcm15207876 (PMC13706132; doi:10.3390/jcm15207876)
Supplement: Supplementary file 1 [file jcm-15-07876-s001.zip › jcm-4573518-supplementary.pdf]

Supplementary Table S1. Baseline laboratory and derived metabolic characteristics according to treatment group

Variable	Overall	Semaglutide	Tirzepatide	Test statistic	p
HbA1c, %	5.75 (5.50–6.10); n=136	5.80 (5.50–6.20); n=70	5.70 (5.40–6.07); n=66	U=2599.0	**0.208**
Fasting insulin, μIU/mL	15.50 (11.80–22.40); n=137	15.10 (10.85–22.35); n=71	16.25 (12.18–23.20); n=66	U=2220.0	**0.598**
Fasting glucose, mg/dL	100.0 (94.0–108.5); n=150	100.0 (93.8–113.0); n=80	100.5 (94.0–105.8); n=70	U=3003.5	**0.444**
HOMA-IR	3.88 (2.77–6.83); n=136	3.88 (2.64–6.93); n=71	3.98 (2.85–6.43); n=65	U=2321.5	**0.953**
Creatinine, mg/dL	0.70 (0.60–0.80); n=127	0.70 (0.60–0.80); n=61	0.70 (0.60–0.80); n=66	U=1955.0	**0.776**
ALT, U/L	24.0 (16.0–33.5); n=119	26.0 (17.5–32.5); n=55	22.5 (16.0–34.0); n=64	U=1836.5	**0.685**
AST, U/L	20.0 (17.0–25.0); n=114	20.0 (17.5–27.0); n=51	20.0 (16.5–24.5); n=63	U=1670.5	**0.717**
GGT, U/L	14.0 (12.0–21.0); n=101	14.0 (12.0–27.0); n=40	14.0 (12.0–19.0); n=61	U=1288.5	**0.636**
CRP, mg/L	3.40 (1.80–6.20); n=116	3.10 (1.75–5.15); n=51	3.80 (1.90–7.20); n=65	U=1462.0	**0.278**
ESR, mm/h	11.0 (7.0–18.0); n=58	10.0 (7.2–17.0); n=22	11.0 (6.8–18.2); n=36	U=377.0	**0.767**
Total cholesterol, mg/dL	202.0 (174.0–234.9); n=61	203.5 (172.0–220.2); n=24	198.0 (181.0–238.0); n=37	U=409.0	**0.610**
LDL cholesterol, mg/dL	137.5 (113.2–165.2); n=140	143.0 (115.5–165.5); n=71	133.0 (109.0–165.0); n=69	U=2665.5	**0.369**
HDL cholesterol, mg/dL	50.5 (44.0–59.2); n=116	49.5 (43.8–59.0); n=52	51.0 (44.0–60.2); n=64	U=1562.0	**0.573**
Triglycerides, mg/dL	122.5 (80.0–155.2); n=136	113.0 (80.0–146.0); n=67	125.0 (95.0–178.0); n=69	U=2014.5	**0.197**
TyG index	8.744 (8.351–9.079); n=132	8.698 (8.195–9.005); n=66	8.841 (8.412–9.131); n=66	U=1919.0	**0.239**

AIP	0.007 (−0.171–0.198); n=111	−0.007 (−0.224–0.172); n=50	0.015 (−0.111–0.207); n=61	U=1368.0	**0.354**

Data are presented as median (Q1–Q3). The available sample size is specified for each variable because laboratory measurements were incomplete in routine clinical records. Treatment groups were compared using the Mann–Whitney U test. Missing observations were excluded on a variable-by-variable basis. HOMA-IR, homeostatic model assessment of insulin resistance; ALT, alanine aminotransferase; AST, aspartate aminotransferase; GGT, gamma-glutamyl transferase; CRP, C-reactive protein; ESR, erythrocyte sedimentation rate; LDL, low-density lipoprotein; HDL, high-density lipoprotein; TyG, triglyceride-glucose index; AIP, atherogenic index of plasma.

Supplementary Table S2. Exploratory changes in laboratory outcomes with limited paired observations at month 3

Variable	n	Baseline	Month 3	Change	W	p
Total cholesterol, mg/dL	7	228.0 (198.0–236.4)	200.0 (175.5–209.5)	−24.0 (−33.0 to −17.0)	0.0	0.016
Non-HDL cholesterol, mg/dL	7	157.0 (140.6–199.0)	150.0 (126.5–178.5)	−18.2 (−24.0 to −13.0)	0.0	0.016
ESR, mm/h	4	14.0 (8.8–17.8)	17.5 (12.5–25.0)	8.5 (−1.8 to 17.8)	1.0	0.500

Data are presented as median (Q1–Q3). Change was calculated as the month 3 value minus the baseline value and is also presented as median (Q1–Q3), not as a confidence interval. Baseline and month 3 values were compared using the Wilcoxon signed-rank test; W denotes the Wilcoxon test statistic. These analyses were based on very small numbers of paired observations and were conducted for descriptive and exploratory purposes only. The nominal p values should not be interpreted as confirmatory evidence, and no adjustment for multiple comparisons was applied. HDL, high-density lipoprotein; ESR, erythrocyte sedimentation rate.

Supplementary Table S3. Sensitivity analysis of model-adjusted percentage body-weight change using categorical follow-up time

Follow-up	Semaglutide, adjusted % change (95% CI)	Tirzepatide, adjusted % change (95% CI)	Adjusted between-group	p

			difference (95% CI)	
Month 1	−3.60 (−4.58 to −2.62)	−4.47 (−5.43 to −3.51)	−0.87 (−2.27 to 0.54)	0.226
Month 3	−8.28 (−9.33 to −7.23)	−12.20 (−13.49 to −10.92)	−3.92 (−5.60 to −2.24)	<0.001
Month 6	−13.45 (−14.85 to −12.05)	−14.84 (−17.94 to −11.73)	−1.39 (−4.81 to 2.03)	0.426

Values are model-adjusted mean percentage changes in body weight with 95% confidence intervals. Negative values indicate weight loss. The adjusted between-group difference was calculated as tirzepatide minus semaglutide; therefore, negative values indicate greater model-estimated weight reduction with tirzepatide. Follow-up time was modeled categorically using the scheduled month 1, month 3, and month 6 visits. The model included treatment group, categorical time, treatment-by-time interactions, age, sex, baseline BMI, and diabetes status, with a patient-specific random intercept. The sensitivity analysis included 245 scheduled follow-up measurements from 148 patients. The overall treatment-by-time interaction was statistically significant (Wald $\chi^2[2]=15.35$, $p<0.001$). BMI, body mass index; CI, confidence interval.

Supplementary Table S4. Sensitivity analysis of model-adjusted percentage body-weight change with additional adjustment for calendar-time treatment initiation

**Follow-up**	**Semaglutide, adjusted % change (95% CI)**	**Tirzepatide, adjusted % change (95% CI)**	**Adjusted between-group difference (95% CI)**	**p**
Month 1	−3.56 (−4.53 to −2.59)	−4.32 (−5.37 to −3.26)	−0.75 (−2.27 to 0.77)	0.330
Month 3	−8.47 (−9.46 to −7.49)	−11.41 (−12.56 to −10.27)	−2.94 (−4.54 to −1.34)	<0.001
Month 6	−14.13 (−15.35 to −12.91)	−15.16 (−18.35 to −11.98)	−1.03 (−4.47 to 2.40)	0.555

Values are model-adjusted mean percentage changes in body weight with 95% confidence intervals. Negative values indicate weight loss. The adjusted between-group difference was calculated as tirzepatide minus semaglutide; therefore, negative values indicate greater model-estimated weight reduction with tirzepatide. Estimates were obtained from a linear mixed-effects model with a patient-specific random intercept. Exact time since treatment initiation was modeled using linear and quadratic terms. The model included treatment group, time, treatment-by-time interactions, age, sex, baseline BMI, diabetes status, and treatment initiation date as a continuous calendar-time covariate expressed in months and centered at the

mean treatment initiation date of the analytic cohort. The analysis included 345 follow-up measurements from 148 patients: 73 initially treated with semaglutide and 75 initially treated with tirzepatide. The overall treatment-by-time interaction remained statistically significant (Wald $\chi^2[2]$=13.39, p=0.001). BMI, body mass index; CI, confidence interval.
